# Effects of Spirulina on Cyclophosphamide-Induced Ovarian Toxicity in Rats: Biochemical and Histomorphometric Evaluation of the Ovary

**DOI:** 10.1155/2013/764262

**Published:** 2013-05-09

**Authors:** Nese Arzu Yener, Orhun Sinanoglu, Erdin Ilter, Aygen Celik, Gulbuz Sezgin, Ahmet Midi, Ugur Deveci, Fehime Aksungar

**Affiliations:** ^1^Maltepe University School of Medicine, Department of Pathology, Maltepe, 34843 Istanbul, Turkey; ^2^Maltepe University School of Medicine, Department of Urology, Maltepe, 34843 Istanbul, Turkey; ^3^Maltepe University School of Medicine, Department of Obstetrics & Gynecology, Maltepe, 34843 Istanbul, Turkey; ^4^Maltepe University School of Medicine, Department of Internal Medicine, Maltepe, 34843 Istanbul, Turkey; ^5^Maltepe University School of Medicine, Department of General Surgery, Maltepe, 34843 Istanbul, Turkey; ^6^Maltepe University School of Medicine, Department of Biochemistry, Maltepe, 34843 Istanbul, Turkey

## Abstract

Cyclophosphamide (Cyc) is known to cause ovotoxicity and infertility in women. Our aim is to investigate the possible ovotoxic effects of Cyc and possible antioxidant and protective effects of blue-green algae, Spirulina (Sp), in rat ovaries. Eighteen rats were given: group I (*n* = 6, control); group II (*n* = 6, CP), a single dose Cyc; group III (*n* = 6, Sp+Cyc), 7 days Sp+single dose Cyc. Tissue malondialdehyde (MDA) levels, superoxide dismutase (SOD), and catalase (CAT) activities are assessed biochemically. Normal and atretic primordial and primary follicle counts for all sections obtained for each ovary are calculated. Mean number of follicle counts for each group are compared. In Sp+Cyc group, tissue MDA levels were significantly lower than those in the CP and higher than those in the C group (CP > Sp+Cyc > C). Tissue SOD activity was significantly higher in Sp+Cyc group than that in the CP group and lower than that in the C group (C > Sp+Cyc > C). No statistically significant difference was found between the ovarian CAT activities in any group. Histomorphometrically, there was also no significant difference between the mean numbers of normal and atretic small follicle counts. Our results suggest that single dose Cyc has adverse effects on oxidant status of the ovaries and Sp has protective effects in Cyc-induced ovotoxicity.

## 1. Background

Cyclophosphamide (Cyc), one of the most effective alkylating agents, is associated with the greatest risk of female infertility [1, 2]. This is mostly attributed to ovarian toxicity and is thought to be strongly related to the cumulative doses of Cyc [1]. Reproductive functions deteriorate by rapid depletion of the oocyte reserve mediated by apoptotic cell death and ovarian atrophy with disappearance of resting primordial follicles [3] and also growing follicles [4] in humans. In other words, apoptosis, which physiologically is an essential event for ovarian function [5] and development of this organ, would become harmful when the ovary is exposed to Cyc [6]. The toxic metabolites of Cyc and the drug itself also interfere with intracellular antioxidation systems which play an important role in detoxifying the reactive oxygen species (ROS) [7]. Superoxide dismutase (SOD), which converts the superoxide anion to hydrogen peroxide, plays a central role in antioxidation reactions [8]. Catalase (CAT), another antioxidant enzyme, catalyzes exclusively the decomposition of hydrogen peroxide to water and oxygen without an electron donor [8]. It is also shown that the lipid peroxidation in ovaries increases in oxidative stress conditions such as ischemia [9]. Biochemical measurement of tissue malonedialdehyde (MDA) levels [9], as a measure of lipid peroxidation and also tissue SOD [9] and CAT [10] enzyme activities have been used to assess oxidative stress/injury in the ovary. On the other hand, the antioxidant supplementation decreases atresia of antral follicles and application of plant extracts that contain antioxidants to scavenge the harmful effects of Cyc attracted the worldwide interest [11]. Spirulina (Sp), a blue-green algae, has been demonstrated as an antioxidant and antiapoptotic in many in vitro and in vivo studies [12]. Its protective effects on the rat ovary against lead-induced [13] and Cyc-induced toxicities has been published [14]. However, no reports are available on the biochemical effects of Cyc on the ovary or the possible protective effects of Sp on ovarian histomorphometry and oxidant status in Cyc-exposed ovaries.

The aim of this study is to define the effect of single-dose Cyc on ovarian small follicle reserve. We also aimed to show any alteration in SOD and CAT activities and also MDA levels in the rat ovary. Finally, we aimed to show the possible protective effect of Sp on Cyc-induced changes in this organ.

## 2. Methods

A fine dark blue-green powder of Hawaiian Spirulina-Arthrospira platensis pacifica (Algbiotek, Istanbul, Turkey) was dissolved in sterile distilled water. Cyc was purchased from Eczacibasi/Baxter Chemical Co. (Istanbul, Turkey). The study was approved (approval number 2011-1) by the Experimental Research Ethics Committee of Maltepe University and was conducted in accordance with European Community Guidelines (EEC Directive of 1986; 86/609/EEC). The dosage and the route of administration of Cyc were determined from that described in the literature [15]. 

### 2.1. Animals and Treatment

The experiment was designed on eighteen Wistar albino rats (180–210 gr) purchased from the Experimental Research Center of Maltepe University. They were randomly put six in each cage under conditions of controlled temperature in individual cages in a room 12L : 12D cycle. Food and water were available ad libitum. After acclimatized for 2 weeks, the experiment was started. Three groups were made with having six rats in each. The control group rats (C) were sacrificed 24 hours after being given a single dose of saline intraperitoneally (ip) (150 mg/kg) on the 8th day of the experiment. The rats in the second group (CP) were sacrified 24 hours after being given a single dose of Cyc, ip (150 mg/kg) on the 8th day of the experiment. The rats in the third group (Sp+Cyc) received Spirulina (1,000 mg/kg bw/day) orally for 7 days and were sacrified 24 hours after being given a single dose of Cyc (150 mg/kg, ip) on the eighth day of the experiment. In the previous literature, Meirow et al. reported that the morphological changes in primordial and primary follicles were observed as early as 24 hours following the exposure of phosphoramide mustard, a toxic metabolite of Cyc [16]. Knowing that the optimal biochemical changes occur in 24–48 hours, we decided to sacrifice the rats at the 24th hour. They were anesthetized with 50 mg/kg ketamine and 10 mg/kg Xylazine before sacrification with exsanguination. In each rat, the right ovary was removed in its entirety, weighed, fixed in 10% formaldehyde, and processed for histomorphometrical evaluation and the whole left ovary was preserved for the biochemical studies.

### 2.2. Histomorphometry

**Figure 1:**
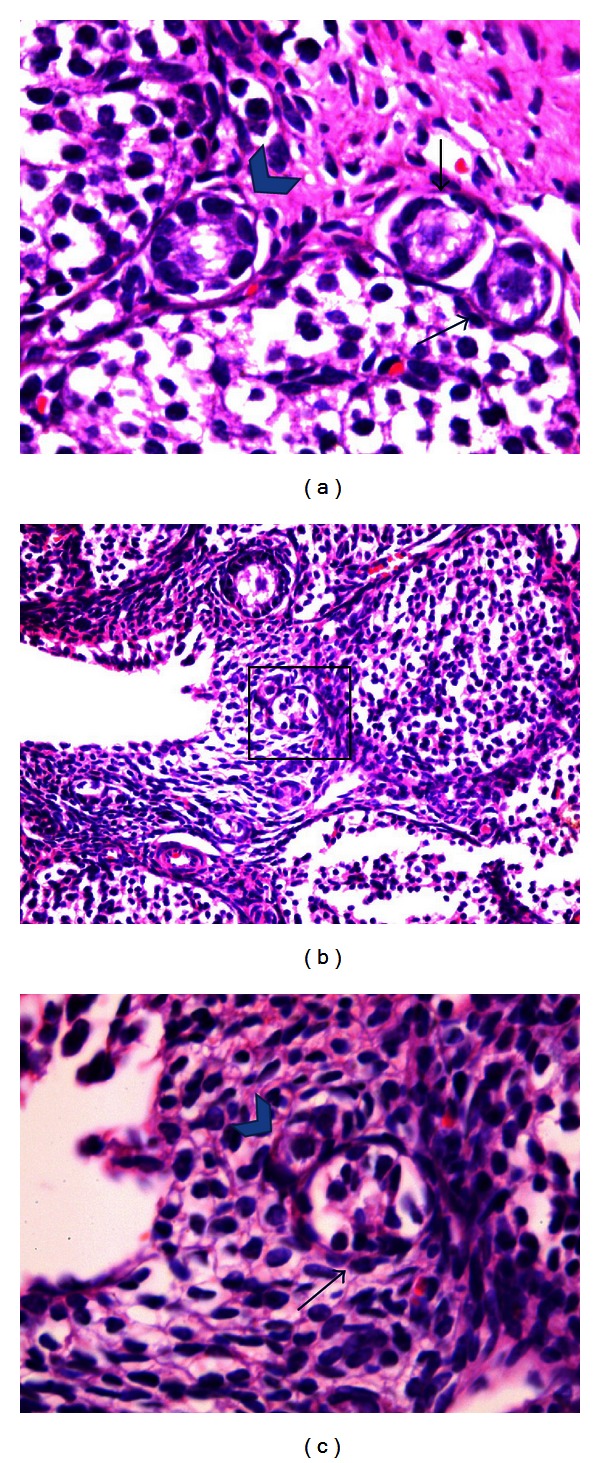
(a) Normal primordial follicle (long arrow), atretic primordial follicle with condensed chromatin (short arrow), and normal primary follicle (arrowhead) (H&E, ×400). (b) Normal primordial follicle (arrowhead) and an atretic primary follicle next to it (arrow) (H&E, ×200). Inset (magnified in (c)) shows an atretic follicle (arrow) with granulosa cells pulled away from the basement membrane (H&E, ×400).

The total small follicle counts, namely, the total primordial and primary follicle counts, were estimated for each ovary. Eight *μ*m sections were prepared and one in each five consecutive sections were taken for this [17]. Approximately sixty slides for each rat ovary were prepared. All small follicles in the 1st, 8th, 16th, 24th, 32nd, 40th, 48th, and 56th sections were counted and the total number of normal primordial follicles (Nprmd), atretic primordial follicles (Aprmd), normal primary follicles (Nprm), and atretic primary follicles (Aprm) for each rat were noted separately [18]. Small follicles were defined as follows [18]. Primordial follicles (prmdf) comprised of an oocyte surrounded by a single layer of spindle-like granulosa cells (Figure 1(a)). Primary follicles (prmf) comprised of an oocyte surrounded by a single layer of cuboidal granulosa cells (Figure 1(a)). Follicles were determined as atretic when they displayed two or more of the following criteria within a single cross section: more than two pyknotic nuclei with condensed chromatin, granulosa cells pulling away from the basement membrane, or uneven granulosa cell layer (Figures 1(b) and 1(c)). Only those follicles in which the nucleus of the oocyte was clearly visible were considered and taken into account [17, 18]. Follicle counting was done manually by one observer (NAY) without having knowledge of the sample identity. We decided not to use any correction factor due to ungoing conflicts in the literature related to it [17, 18].

### 2.3. Tissue 

#### 2.3.1. Homogenization

Fresh tissues were washed with ice cold phosphate buffered saline (PBS) solution (10 mM Na_2_HPO_4_, 10 mM KH_2_PO_4_, 0.9 g NaCl/100 mL, and pH 7.4) and weighed. After the weights were recorded, homogenization was done with a tissue homogenizator (Heidolph DIA×900, Germany) in ice cold PBS immediately (1 mL/mg—volume/weight tissue) and they were kept at −70°C, until assayed.

#### 2.3.2. Measurement of Malondialdehyde (MDA) Level

Samples were thawed and centrifuged. Supernatants were used for the measurements. MDA assay was performed with a spectrophotometric assay (Catalog number NWK-MDA01, Northwest Life Science, Canada). Assay was based on the reaction of MDA with thiobarbituric acid (TBA), forming an MDA-TBA2 complex which absorbs light strongly at 532 nm. The absorbance was directly proportional to the MDA concentration. Intraassay coefficient of variability (CV) was 3.2% and interassay CV was 2.5%. These CV values were taken from the kit inserts. We aimed to show the imprecision rate of the measurement methods and preferred to write them at the end of the method explanation. Data were expressed in nmol of MDA per 1 gram of that tissue.

#### 2.3.3. Measurement of SOD Activity

Homogenates were thawed and centrifuged. SOD activity was measured immediately by a colorimetric assay of SOD (Catalog number NWK-SOD2, Northwest Life Science, Canada). Assay was based on monitoring the autoxidation rate of hematoxylin. In the presence of SOD, the rate of autoxidation was inhibited and the percentage of inhibition was linearly proportional to the amount of SOD present within a specific range. Sample SOD activity was determined by measuring the ratios of autoxidation rates in the presence and absence of the sample. Intraassay and inter-assay CV were 8% and 12%, respectively. Data were expressed as U of SOD per 1 gram of ovary. 

#### 2.3.4. Measurement of CAT Activity

The measurement of the CAT activity in the tissue homogenates was performed with a colorimetric assay. In this assay, the decomposition of peroxide was monitored at 240 nm (Catalog number NWK-Catalase, Northwest Life Science, Canada). The absorbance of hydrogen peroxide at 240 nm was measured directly to calculate the reaction rate since water and oxygen do not absorb at this wavelength. In the presence of CAT, the reaction rate was proportionally enhanced. The intra-assay and the inter-assay CV were 6.12% and 8%, respectively. Data were expressed as U of CAT per 1 gram of ovary.

### 2.4. Statistical Analysis

The total small follicles for all given (1st, 8th,…) sections obtained for each ovary were calculated and noted as the mean number of follicles ±SEM. Differences between groups were analyzed using one-way analysis of variance (ANOVA), and multigroup comparisons were further analyzed by Mann-Whitney *U* test. A value of *P* < 0.05 was considered significant. SPSS 17.0 for Windows, Chicago, Illinois, USA, was used to analyze the data.

## 3. Results

Mean ovarian weight was 0.9 ± 0.09 gr and did not significantly differ between the groups (C: 0.96 ± 0.01; CP: 1.01 ± 0.01; Sp+Cyc: 0.99 ± 0.01) (*P* > 0.005).

**Figure 2:**
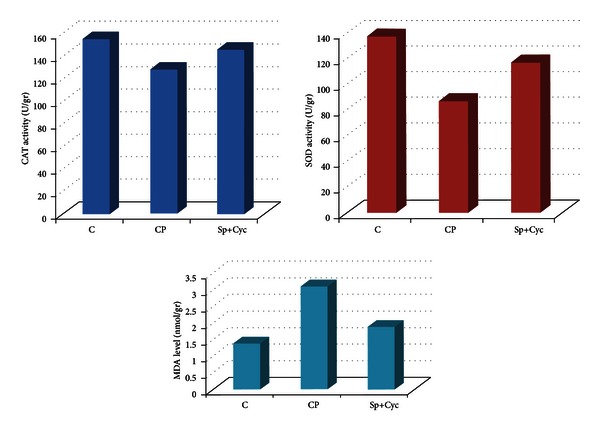
Biochemical analysis of the ovarian CAT, SOD activities, and MDA levels. Values are expressed as mean ± SEM. Statistically significant difference between the groups C and CP for SOD activity and MDA level (*P* < 0.05). No statistically significant difference was found between the groups C and CP for CAT activity and between the groups C and Sp+Cyc for CAT, SOD activities, and MDA levels (*P* > 0.05).

Tissue MDA levels in the Sp+Cyc group were significantly lower than those in the CP group and higher than those in the C group, in ovarian homogenates (CP > Sp+Cyc > C) (*P* < 0.05). Tissue SOD activity was significantly higher in the Sp+Cyc group than that of the CP group and lower than that of the C group in ovarian homogenates (CP < Sp+Cyc < C) (*P* < 0.05). Ovarian CAT levels in the C group were higher than those in the CP group but this was not statistically significant and no significant change was observed between the C and Sp+Cyc groups (C > Sp+Cyc > CP) (*P* > 0.05) (Table 1, Figure 2). Histomorphometrically, there were no significant differences between the mean number of normal and atretic small follicle counts in any groups (*P* > 0.05) (Table 2).

## 4. Discussion

In the present study, the effects of single dose Cyc in the rat ovary were detected, biochemically and histomorphometrically. It was shown biochemically that Sp reversed the adverse effects of Cyc in rat ovaries.

The maintenance of high redox potential is a prerequisite for assuring the reproductive system functions in a healthy organism [8]. Physiologically, ROS are increased in ovary after the preovulatory gonadotrophin surge and also in corpus luteum (CL) during steroidogenesis which involves the cyt P450 system [5, 8]. However, the detoxification of ROS would particularly be important for the oocyte maturation and embryo development [8]. If free radicals are not neutralized by endogenous or exogenous antioxidant molecules such as SOD, then lipid peroxidation would occur at the cell membranes. In these cells, unsaturated lipids converted to peroxides would produce degradation products with toxic aldehyde moieties such as MDA. These subsequently interfere with the ovarian reproductive functions.

The reproductive functions of the ovary are simply assessed with the number of prmdf in the ovarian cortex available to produce viable oocytes at any given time [19]. Although indirect biochemical and ultrasound tests can give an idea about the ovarian follicle density, a more accurate method to evaluate the ovarian capacity is to directly examine a tissue sample containing the follicles [19]. These delicate structures of the ovary are highly susceptible to the chemotherapeutic agents. Cyc is one of these agents and its genotoxicity is shown both experimentally and clinically [6, 14, 20]. It is a prodrug that is activated by cytochrome p450 enzymes to its active metabolites. The latter are responsible for ovarian toxicity. Prmdf count is shown to be adversely effected in high concentrations of phosphoramide mustard (PM), a toxic metabolite of Cyc, both in vitro [20] and in vivo studies [16, 21]. It also destroys the rapidly dividing granulosa cells in antral and secondary follicles in vivo in mice [21, 22] and also the ovarian stromal cells in vivo in rats [23].

Besides these histological and histomorphometric changes, one can also observe biochemical changes like decreased SOD levels, which means that the consumption of this antioxidant enzyme is increased due to Cyc or its metabolites. In other words, oxidation means the overproduction of the free radicals; they covalently bind to DNA and increase the proapoptotic signals [6, 24]. These death signals allow cytochrome c to leak out of mitochondria into the cytosol and then cause the caspase 9 to activate the caspase (cysteine-aspartic acid protease) cascade which then leads to cell death [25]. Caspase 3 is an “effector” enzyme that functions in this cascade to promote the cell death [25]. It is located in the cytoplasm of luteal and theca cells in CL and healthy follicles and also in the granulosa cells of follicles undergoing apoptosis [26]. As a result, Cyc causes all these reactions which deteriorate the ovarian antioxidant status, induce lipid peroxidation, and promote the apoptotic cell death [7].

Although it has not been integrated in the daily medical practice, pretreatment or dietary/pharmacological supplementation with antioxidants may be effective to protect the fertility in women who will undergo chemotherapy [1, 2, 6]. A blue-green algae Spirulina is a powerful antioxidant molecule and is well known for its antioxidant, antiapoptotic properties [12]. It is composed primarily of various components such as B-complex vitamins, chlorophyll, *β*-carotene, vitamin E, superoxide dismutase, and numerous minerals [12]. C-phycocyanin, a protein-bound pigment found in Sp, inhibits oxalate-mediated lipid peroxidation and prevents injury in many tissues. Its protective effects on gonads are also studied [14]. In that study, Chamarro-Cevallos et al. showed in mice that Sp reversed the postimplantation losses caused by Cyc. Sp pretreatment even at low doses was shown to prevent the Cyc-induced semen abnormalities [14].

In the present study, the parameters of oxidative stress, that is, MDA was found as markedly increased and the activity of SOD was markedly decreased in the ovary of Cyc-treated rats suggesting that Cyc treatment caused oxidative damage to the lipids and proteins in this organ. The rat ovaries in the Sp+Cyc group had significantly increased SOD levels and decreased MDA levels suggesting that Sp protects against the adverse effects of Cyc.

We think that the antioxidants in Sp, mainly C-phycocyanin, SOD, B-complex vitamins, chlorophyl, *β*-carotene, and vitamin E [12], may act synergistically to restore the antioxidant status of the ovary. These compounds probably reduce the formation of potent oxidant peroxynitrite which is produced by the reaction of nitric oxide with superoxide anion by scavenging the superoxide anion with SOD activity. There were no significant changes in either CAT levels or small follicle counts among all groups (*P* > 0.05). This may be due in part to our measurement of nonselective CAT enzyme which uses hydrogen peroxide. Hydrogen peroxide can be removed not just by CAT, but also by other components of the antioxidant system, such as glutathione peroxidase, peroxiredoxin, and glutathione S-transferase as well [27]. We did not notice any significant change in ovarian weights as was previously reported in the ovaries exposed to Cyc [24].

One limitation of our study is that we did not study the growing follicles which are highly susceptible to Cyc effects [24]. For this, each rat should have been sacrified in its proestrous phase to estimate the total secondary and the antral follicle numbers. Instead, we focused on the changes in the oxidant status of the ovary both with Cyc and Sp after a certain period of time. So it was infeasible to study growing follicles and it may be a subject of a future study.

One puzzling point for this study is based on a longstanding debate about whether the antioxidants are used together with anticancer drugs since the former might protect cancer cells from treatment modalities. Some studies based on randomized clinical trials support this opinion [28] whereas others show that the antioxidants are not protecting the cancerous cells from the chemotherapeutic agents but rather enhancing their killing power [29]. We think that this controversial subject is beyond the scope of our study and should be discussed in a future study.

To the best of our knowledge, we have shown for the first time, based on biochemical and histomorphometrical findings, that a single dose (150 mg/kg) Cyc induces the lipid peroxidation and the oxidant status in the rat ovary and it does not affect the ovarian small follicle counts in rats. Pretreatment with Sp attenuates Cyc-induced lipid peroxidation and increases the SOD levels in the rat ovary suggesting that it may be effective in protecting the Cyc-exposed tissues in human. Sp does not affect the small follicle counts either adversely or favorably. Possible molecular mechanisms for Cyc induce the apoptosis signaling pathways by regulating the ROS-mediated pathways, and the role of certain genes in apoptosis mechanisms, especially in higher or multiple doses of Cyc, should certainly be the subject of future studies.

## Figures and Tables

**Table 1:** Biochemical analysis of the ovarian CAT, SOD activities, and MDA levels.

	CAT (U/gr)	SOD (U/gr)	MDA (nmol/gr)
C (#6)	155.35 ± 25.02	137.88 ± 21.33	1.39 ± 1.02
CP (#6)	128.74 ± 11.48^b^	87.86 ± 15.21^a^	3.10 ± 0.86^a^
Sp+Cyc (#6)	146.04 ± 22.07^b^	117.45 ± 24.79^b^	1.90 ± 0.72^b^

CAT: catalase, SOD: superoxide dismutase, MDA: malonedialdehyde, C: control group, CP: cyclophosphamide group, Sp+Cyc: Spirulina+cyclophosphamide group. Values are expressed as mean ± SEM. ^a^Statistically significant difference between the groups C and CP for SOD activity and MDA level (*P* < 0.05). ^b^No statistically significant difference between the groups C and CP for CAT activity and between the groups C and Sp+Cyc for CAT, SOD activities, and MDA levels (*P* > 0.05).

**Table 2:** Analysis of ovarian follicle counts in all groups (mean of number of counts of given follicle types per ovary for each group).

Groups	Nprmd	Aprmd	Nprm	Aprm
C (#6)	22.0 ± 5.6	6.50 ± 1.4	10.3 ± 2.8	3.5 ± 1.8
CP (#6)	20.5 ± 3.3	12.2 ± 3.5	6.2 ± 1.2	5.8 ± 2.3
Sp+Cyc (#6)	23.5 ± 3.2	5.8 ± 1.2	14.17 ± 4.2	3.50 ± 1.1

C: control group, CP: cyclophosphamide group, Sp+Cyc: Spirulina+cyclophosphamide group, Nprmd: normal primordial follicle count, Aprmd: atretic primordial follicle count, Nprm: normal primary follicle count, Aprm: atretic primary follicle count. Values are expressed as mean ± SEM. No statistically significant difference was found between the groups C and CP; or between CP and Sp+Cyc (*P* > 0.05).
